# Is There Still a Role for Autologous Stem Cell Transplantation for the Treatment of Acute Myeloid Leukemia?

**DOI:** 10.3390/cancers12010059

**Published:** 2019-12-24

**Authors:** Felicetto Ferrara, Alessandra Picardi

**Affiliations:** 1 Division of Hematology and Stem Cell Transplantation Program, AORN Cardarelli Hospital, 80128 Naples, Italy; alessandra.picardi@aocardarelli.it; 2 Department of Biomedicine and Prevention, University of Tor Vergata, 00119 Rome, Italy

**Keywords:** acute myeloid leukemia, autologous transplantation, post-remission therapy, minimal residual disease

## Abstract

After intensive induction chemotherapy and complete remission achievement, patients with acute myeloid leukemia (AML) are candidates to receive either high-dose cytarabine-based regimens, or autologous (ASCT) or allogeneic (allo-SCT) hematopoietic stem cell transplantations as consolidation treatment. Pretreatment risk classification represents a determinant key of type and intensity of post-remission therapy. Current evidence indicates that allo-SCT represents the treatment of choice for high and intermediate risk patients if clinically eligible, and its use is favored by increasing availability of unrelated or haploidentical donors. On the contrary, the adoption of ASCT is progressively declining, although numerous studies indicate that in favorable risk AML the relapse rate is lower after ASCT than chemotherapy. In addition, the burden of supportive therapy and hospitalization favors ASCT. In this review, we summarize current indications (if any) to ASCT on the basis of molecular genetics at diagnosis and minimal residual disease evaluation after induction/consolidation phase. Finally, we critically discuss the role of ASCT in older patients with AML and acute promyelocytic leukemia.

## 1. Introduction

Acute myeloid leukemia (AML) is a complex, genetically heterogeneous disease of hematopoietic stem cells (HSCs), characterized by multiple somatically acquired driver mutations, co-existing competing clones and disease evolution over time [1,2,3]. The accumulation of genetic changes results in the disruption of normal mechanisms of HSC self-renewal, proliferation and differentiation, and leads to the accumulation of immature cells (myeloblast) in the bone marrow and peripheral blood. The advent of high-throughput new molecular techniques has provided new insights into the molecular basis of the disease, so that different genetic profiles at diagnosis can definitely influence the therapeutic strategy either at front-line or after relapse [4,5,6,7]. Furthermore, genetic abnormalities are powerful prognostic factors in both young and elderly patients with AML [8,9], and the European leukemia Net (ELN) panel have proposed a widely adopted classification system with three risk groups (favorable, intermediate and adverse), which is especially valuable for the selection of post complete remission (post CR) therapy [10]. 

## 2. Post Remission Therapy

**Figure 1:**
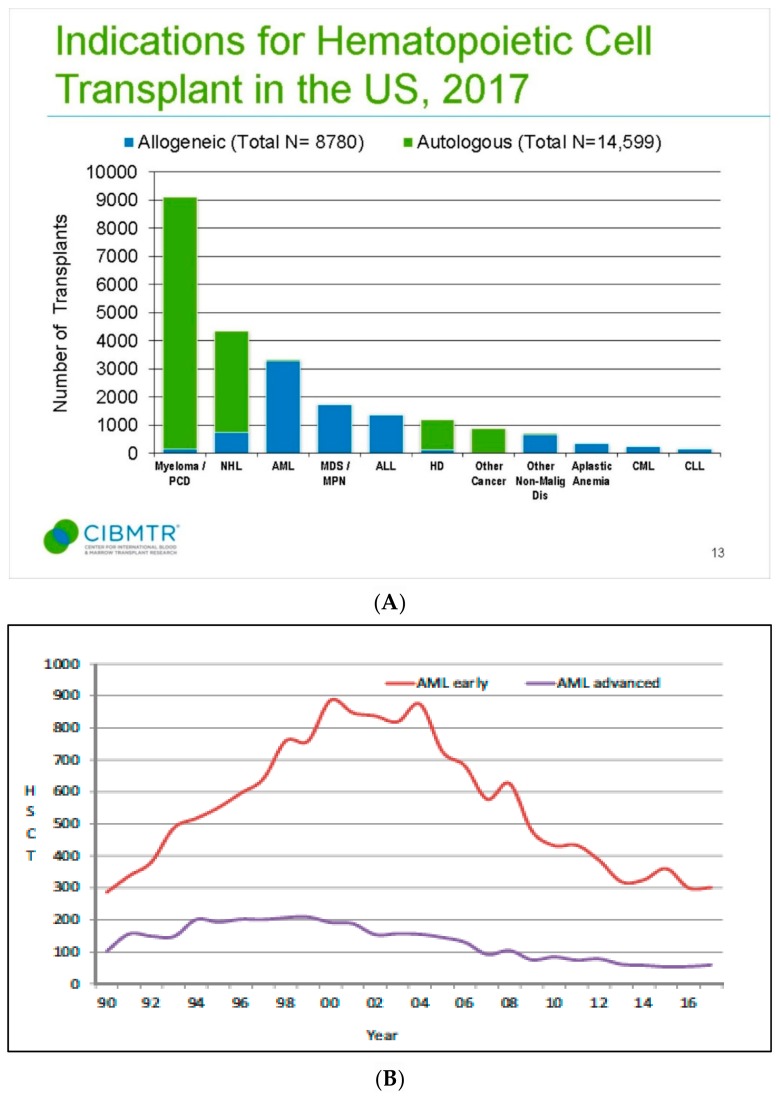
Declining rates of autologous stem cell transplantation (ASCT) in acute myeloid leukemia (AML) in the USA (panel (**A**), accessed at www.CIBMTR.org) and Europe (panel (**B**) [27]).

**Figure 2:**
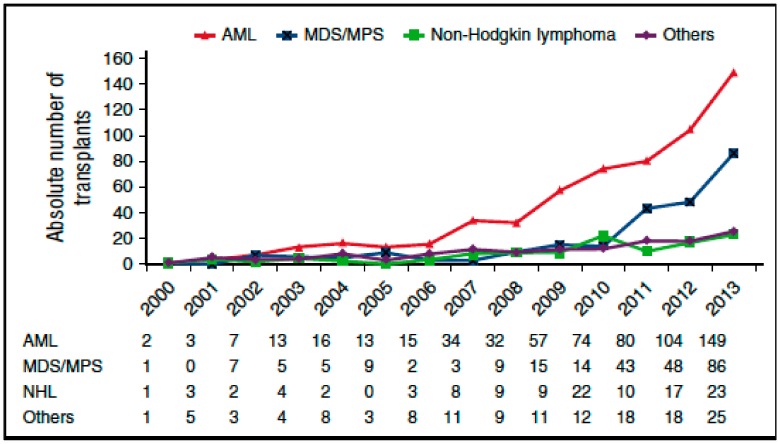
Annual number of allogeneic hematopoietic stem cell transplantations (allo-SCTs) in patients aged 70 years and older by indication: major increase in AML [30,31].

**Figure 3:**
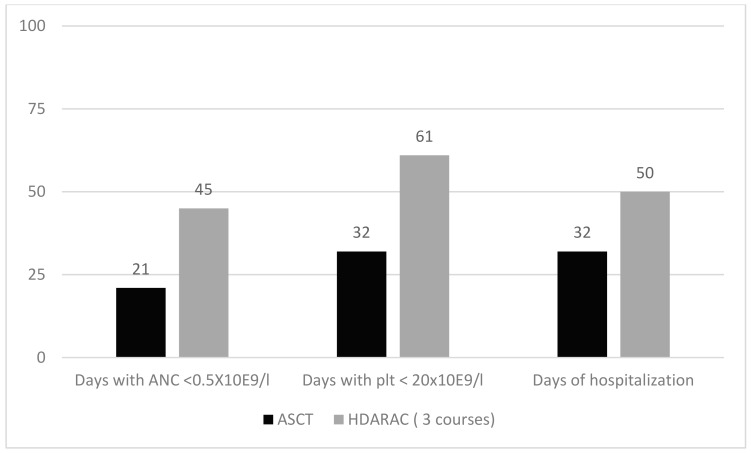
Comparison of days of neutropenia, thrombocytopenia and hospitalization between ASCT and three courses of a high dose of cytarabine (HDARAC); all differences are statistically significant (*p* < 0.01).

The selection of optimal post-CR therapeutic option represents a crucial step for preventing relapse, in that in most patients in morphological CR after induction therapy, different amounts of minimal residual disease (MRD) are still present [11,12]. Post CR treatment of AML includes in all eligible patients, one consolidation course, based on high/intermediate dose of cytarabine (HDARAC/IDARAC), followed by two or three cycles of similar chemotherapy; alternative options include autologous stem cell transplantation (ASCT) and allogeneic SCT [13]. Currently, the role of allogeneic SCT is clearly defined, whatever the donor, for patients defined as being at high biological risk, according to the either ELN [10] or National Comprehensive Cancer Network (NCNN) criteria [14], for secondary AML, or for patients who fail to achieve CR after the first induction therapy. In this unfavorable setting, alternative strategies based on either ID, or HDARAC or ASCT, may have a role only in patients with high risk of transplant related mortality. On the other hand, for patients with core-binding-factor AML, i.e., AML with t(8;21); AML with inv(16) or t(16;16); and an *NPM1* mutation in absence of an *FLT3* mutation or with *FLT3* low allelic burden, ELN recommends, after induction, up to four courses of ID/HDARAC, even though it remains unclear whether ID or HDARAC of two or three course would be preferred [15,16]. Numerous studies have demonstrated that ASCT could be offered to younger patients in the favorable and intermediate cytogenetic risk groups with results not inferior to ID/HDARAC in terms of relapse occurrence and survival [17,18,19,20,21,22,23,24,25]. Relapse remains a major cause of treatment failure ASCT, and in most cases it occurs within the first 2 years after transplantation. In order to assess the clinical outcome and risk factors for patients surviving in CR after two years, 3567 adult patients (median age, 45 years) with AML who underwent autografting in CR1 (86%) or CR2 (14%) between 1990 and 2008 were retrospectively analyzed [23]; 32% received bone marrow and 68% peripheral blood stem cells (PBSCs), with a median follow-up of 6.9 years. While at 5 and 10 years after ASCT, the probability of leukemia-free survival was 86% and 76%, respectively, it should be considered that the above data refer to patients surviving and being free of disease recurrence at least 2 years after ASCT; furthermore, patients with secondary AML were excluded from the analysis. The incidence of relapse was relatively low (11% at 5 years and 16% at 10 years, respectively), while non relapse related mortality occurred in 3% and 8%, respectively. Of interest, the survival was decreased compared with the expected survival of the general European population. In a multivariate analysis, decreased probability of leukemia free survival was associated with use of PBSC and older age, while nonrecurrence mortality was found to be affected by older age only. While the results of this analysis show that late recurrences remain a major concern after autologous stem cell transplantation among patients with AML, they also indicate that a considerable proportion of AML patients receiving ASCT have definitively been cured. In this regard, however, positive selection bias, depending on successful stem cell harvest and exclusion of patients with early relapse, should be taken into account. The possibility of individualized prediction of leukemia-free-survival (LFS) after ASCT was recently investigated. In more detail, Shouval et al. developed a prediction model for LFS from a cohort of 956 patients with de novo AML autografted in CR1 [17]. In this registry study, a multivariate Cox regression modeling with backward selection was used to select variables for the construction of the nomogram. Age and cytogenetic risk (with or without FMS-like tyrosine kinase three internal tandem duplications) were predictive of LFS and were used for the construction of a nomogram. Each factor in the nomogram was ascribed points according to its predictive weight. Through the calculation of the total score, the probability of LFS at 1, 3 and 5 years for each patient could be estimated with excellent discrimination. Overall, patients were stratified into four incremental 5-year prognostic groups, with the probabilities of LFS and overall survival ranging from 25% to 64% and from 33% to 79%, respectively. Worthy of note in that study is that the impact of total body irradiation was analyzed and no impact was demonstrated on LFS. The authors concluded that the Auto-AML nomogram score is a reliable tool integrating individual prognostic factors in order to provide a probabilistic estimation of LFS after ASCT for patients with AM [17]. Notwithstanding, either European Bone Marrow Registry (EBMT) or Center for international blood and marrow research (CIBMTR) data clearly demonstrate that the adoption of this procedure has been declining in the last years [26,27], as shown in Figure 1 and Figure 2. Apart from logistic issues which are related to collection and cryopreservation of stem cells and the possibility of inducing infertility in younger patients, a major issue in the field of ASCT is related to the possibility of achieving reassuring data on MRD in the graft and the patient. Finally, the progressive adoption of alternative donors in the setting of haploidentical transplant and the increase of age upper limit for allogeneic hematopoietic stem cell transplantations (allo-SCT), have, in turn, reduced the use of ASCT for AML, as shown in Figure 3 [28,29,30,31]. In Table 1, we summarize main factors related to the declining use of ASCT in AML in the last decade. 

## 3. Factors Related to the Appropriate Use of ASCT

### Molecular Genetics

The rationale for the use of ASCT in AML as in other hematologic malignancies, such as Hodgkin and non-Hodgkin lymphoma, relies on the possibility of eradication of the malignant clone by high dose chemotherapy with stem cell support. Therefore, only selected patients with chemosensitive disease can take advantage of ASCT. Among these, patients Core-Binding-Factor (CBF) AML and AML with a *NMP1* mutation in absence of an *FLT3* one are the ideal candidates to receive ASCT [32,33,34]. Notwithstanding, even in this setting, the role of ASCT remains unclear, although most studies that have compared ASCT with intensive consolidation chemotherapy (ICC) demonstrated a significantly lower rate of relapse following ASCT [17,18,19,20,21,22]. However, results in terms of survival were less encouraging because of transplant-related deaths and the low rate of second CR in patients who relapsed after ASCT; therefore, in the last year, ASCT has become less popular both in Europe and the USA. As a matter of fact, neither NCNN [14] nor ELN [10] guidelines recommend ID/HDARAC instead of ASCT in low-risk AML. On the contrary, ELN allows one to consider ASCT for patients with intermediate or adverse genetics, provided that they are MRD negative and with a high risk of treatment related mortality (TRM) in CR1. In this regard, we want to point out that toxicity and mortality related to ASCT have greatly decreased since the use of peripheral-blood stem cells was introduced, even in older patients. In addition, reduction of relapse rate, which has been more frequently reported with ASCT than chemotherapy, would represent a main therapeutic objective in the therapy of AML, just as it is for any malignant disorder. Finally, consolidation therapy based on repeated courses of ID/HDARAC is probably more toxic and difficult to give than ASCT, and is poorly feasible in patients aged over 55–60 years or more. In our experience, days of hospitalization and neutropenia as well as the number of hospital admissions were consistently longer after three courses of ID/HDARAC compared to ASCT (Figure 3). The clinical value of ASCT in comparison to allo-SCT or intensive chemotherapy (ICT) was also investigated in the favorable AML subgroup with double mutant CEBPA (CEBPAdm) in a series of 124 patients treated in four trials in Europe. In this subgroup of favorable AML patients, relapse free survival was lower for those proceeding to allo or auto-HSCT in CR1 compared with chemotherapy (*p* < 0.001), whereas only a trend was reported as to overall survival (*p* < 0.12). Of interest, among relapsed patients, 83% achieved a second CR and most of them received an allo-SCT. Survival of relapsed patients measured from date of relapse was 46% after 3 years. The conclusion of the authors was that adult AML patients with CEBPAdm benefit from allo-SCT and ASCT; relapsed patients still had a favorable outcome after reinduction followed by allo-SCT [35].

## 4. Minimal Residual Disease: What We Know and What We Do Not Know

The possibility of defining residual disease far below the morphological level of 5% blast cells is changing the landscape of response evaluation and risk classification in AML [12,36,37,38]. Real-time quantitative PCR (qPCR) and multiparameter flow cytometry (MFC) are effective techniques for monitoring MRD before and after ASCT in patients with AML, and MRD status pre-ASCT is an independent prognostic factor for both OS and RFS after ASCT [12,36,37,38]. Availability of the newer and more sensitive technology to quantify the level of leukemic burden has definitively favored the use of MRD in AML; in particular, in selected genetic subtypes, such as CBF-AML and NPM1, mutated AML, molecular monitoring of patients in morphologic CR allows identification of those patients deemed at high risk for disease relapse, ultimately resulting in a further improvement of clinical outcome [39,40]. Furthermore, within these cytogenetic subgroups of AML, identifying the best timepoints for MRD examination is also very important for applying MRD-directed risk stratification treatment. Finally, not only MRD level but also the kinetics of MRD are reliable for the prediction of imminent relapse [41,42]. Therefore, the response category CR_MRD-_ has been proposed by ELN panel, because relapse is more likely in patients in CR or CR with incomplete hematologic recovery (CRi) and detectable residual disease [10]. The best time to test for MRD in patients in CR by conventional criteria is not settled [43]. Assessment of MRD after cycle 2 or even cycle 1 of induction allows earlier identification of poor responders. However, MRD can disappear after consolidation therapy [44]. The frequency with which this occurs may differ in different molecular subsets and a future assessment of these frequencies will likely inform therapeutic decisions. Overall, the assumption that in AML, MRD negativity, either in the patient bone marrow or in the graft, represents an essential prerequisite for considering ASCT, is quite appropriate. 

A recent trial was conducted by the GIMEMA group in which post-remission therapy of young patients with de novo AML combined cytogenetics/genetics and post-consolidation levels of MRD [45]. After induction and consolidation, favorable-risk patients were programmed to receive ASC; poor-risk patients allo-SCT; and intermediate-risk patients auto or allo-SCT depending on the postconsolidation levels of MRD. Overall, 110/177 (62%) and 130/188 (71%) ASCT or allo-SCT candidates received it, respectively. Two year overall (OS) and disease-free survival (DFS) of the whole series was 56% and 54%, respectively. Two-year OS and DFS were 74% and 61% in the good risk category; 42% and 45% in the adverse risk category; 79% and 61% in the intermediate risk MRD-negative category; and 70% and 67% in the intermediate risk MRD-positive category. The authors concluded that ASCT may still have a role in favorable and intermediate risk, MRD-negative categories. This study, in spite of some limitations due to the changes occurring over the time, such as more modern biologic knowledge, new AML classifications and an ever more frequent and effective molecular MRD monitoring represents a remarkable attempt to apply a prospective program of risk-adapted, MRD-driven therapy, integrating upfront genetics and post-consolidation MRD status, in AML of adults. These data confirm the original recommendations regarding ASCT, which use should be limited to chemosensitive patients who have achieved MRD-negative status in the marrow and leukapheresis products. Technological advances for detection of MRD (not only in AML) include the use of digital droplet PCR, or next-generation sequencing (NGS). In particular, molecular MRD detection by NGS can be successfully applied to most newly diagnosed AML patient, in whom multiple molecular aberrations are detectable. However, detection based on NGS must overcome different challenges before its introduction into clinical practice. In particular, limitations of NGS-based methodology for the assessment of MRD are still related to still limited sensitivity and specificity, and to inability, in selected cases, for discrimination between residual leukemia and clonal hematopoiesis. Finally, it is mandatory to use standardized techniques, apply rigorous bioinformatic analyses and define specific time-points, developed in the context of laboratory networks. In this regard, the ELN MRD Working Group is currently aiming to improve and harmonize methodologies for NGS-based detection of MRD in AML [46,47].

## 5. Conditioning Regimens

In the ASCT setting, the conditioning regimen would play a key role due to the double aim of space’s creation and disease eradication. The historical conditioning regimen is represented by the “Tutschka regimen” [48], based on the combination of oral busulfan 16 mg/kg with cyclophosphamide 120 mg/kg (BUCY). However, two recent retrospective studies, on behalf of the Italian Group for Blood and Marrow Transplantation and European Society for Blood and Marrow Transplantation (EBMT), respectively, have suggested that the association of busulfan with high-dose of melphalan (BUMEL) may led to improved outcomes in AML [49,50]. Furtherly, in 2017 Gorin et al. [51] reported a retrospective multicenter analysis, from the EBMT database, comparing BUMEL to BUCY as preparative regimens before ASCT for patients with AML in complete remission (CR). This latter study included a total of 853 patients, of whom 596 received BUCY and 257 received BUMEL with a median follow-up for survivors of 44.2 months (range, 1.08–127.8 months) and 50.33 months (range, 1.57–124.46 months), respectively. Of note, this study showed a lower relapse rate (39.5% versus 52%, *p* = 0.003), a better LFS (55.4% versus 44.6%; *p* = 0.005) and a better OS (73.8% versus 63; *p* = 0.0007) for patients who underwent BUMEL as a conditioning regimen, whereas the NRM was similar (*p* = 0.66) in the two groups. We pioneered the use of idarubicin given as continuous infusion in combination with oral or intravenous busulfan [52]. Using the same regimen, researchers in Nanjing reported more recently, on 32 AML patients who underwent ASCT in CR1, with only one non-relapse death. Median OS and LFS were not reached at 30 months in this series [53]. The uniqueness of this regimen lies with the specific use of high dose IDA, in combination with classical dose of oral Bu and the removal of cyclophoshamide (Cy), which is not included in induction, consolidation or salvage treatment for AML, and, therefore, would exert minimal effect on leukemic population. More recently, we replaced oral with intravenous Bu, achieving substantial reduction of nonhematologic toxicity, namely, oral mucositis [54]. Best results were achieved in AML with NPM1 mutation in absence of FLT3 and CBF-AML, and the regimen was also feasible in an older patient population [55]. However, no prospective randomized studies of conditioning regimens’ comparison before ASCT in AML patients are available, and, in our opinion, the definition of the ideal regimen still represents an interesting field of investigation. 

## 6. Stem Cell Source

Based on the rationale that after two chemotherapy cycles the leukemic burden decreases at least 2 logs with respect to its onset, the best timing to collect the stem cells would fall after the first consolidation cycle. Although, some reports suggested higher incidence of relapse with peripheral blood rather than marrow as a source of stem cells in adults with AML autografted in CR1 [56], nowadays, the preferred stem cell source is represented by te peripheral blood stem cells (PBSCs) collected after consolidation therapy plus stimulation with granulocyte colony stimulating factor (G-CSF), in MRD negative patients. The main advantages of PBSCs as a stem cell source are the faster neutrophil and platelet recovery times than bone marrow, with consequently reduced infection, bleeding and hospitalization risks. The PBSC target dose is considered an amount of CD34+ cells ≥ 2 × 10^6^/kg body weight. If these numbers are not met after 4–5 attempts of collection, the patient is considered as a failure for mobilization and the availability of a bone marrow harvest unit can be considered, even though in a considerable number of cases bone marrow collection is also likely to fail with regard to reaching the target CD34 cell dose. In the case of a bone marrow harvest, the target cell dose is 2 × 10^6^/kg CD34 positive cells, or 2 × 10^8^/kg nucleated cells. In the case of a poor PBSC collection, followed by a poor BM harvest, the use of the combination of the two stem cell sources should be considered. According to JACIE standard, regardless of the stem cell source, the viability of the attached segment to the stem cell bags before starting the conditioning regimen is mandatory in order to assess the safety of the autologous product to guarantee the hematopoietic recovery after the transplant procedure [57]. Summarizing, the best collection option should include the MRD assessment after induction/consolidation followed by PBSC mobilization. 

## 7. ASCT in Older Patients with AML

Prospective studies in older AML patients assessing the benefits of ASCT compared to chemotherapy consolidation or allogeneic transplantation are lacking. In older patients, different biological and clinical factors account for poor prognosis. The former include leukemic cell resistance related to more frequent adverse mutation pattern and karyotype and preferential involvement of chemorefractory, early hemopoietic precursors in the pathogenesis of the disease; the latter are represented by frequent comorbidities, that can induce reluctance by clinicians to treat unfit patients [58,59,60]. Higher incidence of AML secondary to previous myelodysplastic syndrome (MDS) further accounts for poor outcomse [61,62]. With the aim of offering a potentially curative approach to elderly AML patients as well, in the recent years, numerous studies have been conducted to explore the feasibility and safety of allo-SCT in advanced age. Results are extremely encouraging, and the upper age limit is increasing up to 75 years [30,31]. On the other hand, poor feasibility of the administration of repeated courses of HDARAC in older AML patients was clearly demonstrated since the publication of a pivotal study by the Cancer and Leukemia Group B in 1994 [63]. In daily practice, either one or two courses of IDARAC are usually preferred, in absence of data deriving from controlled randomized trials, as far as the more effective number is concerned. As in young adult patients, ARAC consolidation is unable to eradicate leukemic clone in the subgroups of patients with unfavorable molecular genetics at diagnosis, particularly if they remain MRD positive. Accordingly, these patients, if clinically eligible, should undergo allo-SCT as soon as possible after initial treatment with ICT or hypomethylating agents, which can in turn be employed as bridge to transplant. However, allo-SCT over the age of 65 years can be currently offered to no more than 20%–25% of the patients, because of different reasons, including low CR rate, early relapse and poor performance status after induction/consolidation therapy. A number of studies have definitively demonstrated that ASCT is feasible and safe in older patients with AML [52,64]. It is conceivable that concerns related to molecular findings at diagnosis and to MRD are relevant, as in young adults. Given that in advanced age the incidence of AML with favorable AML criteria accounts for no more than 15%–20% of patients, the use of ASCT is in turn limited to a minority them. An essential prerequisite to performing ASCT is represented by successful mobilization and collection of an adequate number of PBNSCs. Risk factors for poor mobilization in AML patients involve stem cell senescence, clonal hemopoiesis of indeterminate potential, loss or dysfunction of the stem cell niche and the presence of previous undiagnosed myelodysplastic syndrome (MDS). In a retrospective series of 40 patients, we previously demonstrated that age has no influence on PBSC mobilization and collection in AML; in most patients, PBSCs were collected after consolidation based on continuous sequential infusion of fludarabine and ARAC, followed by granulocyte-colony-stimulating factor with a successful rate over 90% [65]. Overall, results in terms of survival after conditioning with Bu and Ida were promising either in de novo or secondary disease, but relapse rate in patients with unfavorable cytogenetics was impressively high [66]. In patients with mobilization failure, harvesting HSCs from bone marrow could be considered, but in our own experience, it is poorly accepted by patients and associated with different inconveniences; therefore, we do not currently consider bone marrow harvesting, and, in the case of no or insufficient mobilization, we administer one further course of IDARAC. In conclusion, in older patients with AML, ASCT can be safely offered to the subgroup with *CBF* and *NPM1* mutations in the absence of an *FLT3* mutation, provided that an adequate number of PBSCs are collected. For intermediate or high risk patients, the procedure would be considered in controlled trials, including new agents in different phases of the therapeutic program. 

## 8. ASCT in Acute Promyelocytic Leukemia

Acute promyelocytic leukemia (APL) is a highly curable disease, since the introduction of all-trans-retinoic acid (ATRA) and arsenic trioxide (ATO). The advent of ATRA and its inclusion in combinatorial regimens with anthracycline chemotherapy has provided cure rates exceeding 80%; however, this widely adopted approach also induces significant toxicity, including severe myelosuppression and occasional occurrence of secondary leukemias. More recently, the advent of ATO and its use in association with ATRA has further improved the outcome of low-risk patients with cure rate higher than 90%. The combination of ATRA and anthracyclines remains the gold standard for high risk patients, but in this setting as well, the probability of curing exceeds 80%. Accordingly, there is not a role for stem cell transplantation in APL in CR1, independently from any initial risk category [67]. On the contrary, either auto or allo-SCT may have indications in relapsed patients. Current recommendations from an expert panel of ELN suggested that patients who relapsed after ATRA plus chemotherapy should be treated with an ATRA plus ATO based approach as salvage therapy until achievement of MRD negativity, whereas for those relapsing after ATRA plus ATO, an ATRA-plus-chemotherapy approach could be the most appropriate option [68]. A potential exception for crossing over to a different treatment for patients who relapsed may be considered for those with late relapse (e.g., >2 years in CR). The achievement of second molecular CR would represent a bridge to stem cell transplantation. Some years ago, we suggested that ASCT could be of benefit in APL relapsed patients, if performed with a molecularly negative graft in second molecular CR offering a valid chance for achieving a cure [69]. Such an approach should also be considered in relapsed patients with an HLA-compatible donor; namely, in those with a first CR lasting more than two years, or in unfit or elderly individuals. More recently, ELN recommendation suggested that ASCT should be considered the first choice for eligible patients achieving second molecular remission, even though a report from United Kingdom questions the role of transplantation, at least in patients achieving molecular remission with ATO and ATRA who do not have CNS disease at relapse and who have received a full course of consolidation with ATO [70]. The role of allo-SCT would be limited to patients failing to achieve molecular remission who are candidates for allogeneic HSCT. 

## 9. Conclusions

The selection of the most appropriate post remission approach in AML is still the object of investigation and new opportunities will be available in the next future available because of the introduction of different new agents in the daily practice. In particular, it is of utmost importance to identify patient categories in whom conventional chemotherapy is not appropriate because of intrinsic resistance of leukemic clone. Allo-SCT is regarded as a curative strategy for AML and must be offered to high risk patients; the efficacy of ASCT remains controversial; in particular, its superiority in comparison to consolidation chemotherapy with repeated courses of ID/HDARAC in patients with good risk disease is still matter of debate, although most studies account for lower relapse rate after ASCT. Nowadays, ASCT is not considered a standard treatment for AML in the United States, while it is still somewhat considered in Europe and Japan [71], even if its use is progressively declining. It is our opinion that ASCT in AML does still represent a valid therapeutic option for AML patients at low risk in CR1, provided that MRD negativity is achieved. Improvement in the detection of MRD with more sophisticated techniques, such as next generation sequencing analysis, would result in further improvements of the selection of best candidates for ASCT and in the ideal monitoring of patients while in CR. Finally, the development of new targeted therapy for AML could represent an important option for a molecularly driven post-ASCT therapy [72,73]. 

## Figures and Tables

**Table 1:** Factors related to the declining use of autologous transplantation in AML.

Lack of definitive evidence concerning survival advantage
Logistic reasons related to stem cell collection and cryopreservation
Lack of reassuring data on minimal residual disease in the marrow and graft
Poor results at relapse after ASCT as compared to chemotherapy
Increasing number of matched unrelated and haploidentical donors for allo-sct
Feasibility of allo-SCT in increasing percentage of older patients
